# Approaches in the Treatment of Cesarean Scar Pregnancy and Risk Factors for Intraoperative Hemorrhage: A Retrospective Study

**DOI:** 10.3389/fmed.2021.682368

**Published:** 2021-06-24

**Authors:** Yaying Lin, Chang Xiong, Chunlin Dong, Jinjin Yu

**Affiliations:** ^1^Department of Obstetrics and Gynecology, Affiliated Hospital of Jiangnan University, Wuxi, China; ^2^Wuxi Medical College, Jiangnan University, Wuxi, China

**Keywords:** cesarean scar pregnancy, hysteroscopic curettage, uterine artery embolization, clinical classification, intraoperative hemorrhage

## Abstract

**Background:** Cesarean scar pregnancy (CSP) involves a rare form of placental attachment that often leads to life-threatening conditions. The best treatment for CSP has been debated for decades. We aimed to evaluate the different treatments for CSP and analyzed the risk factors for intraoperative hemorrhage.

**Methods:** CSP patients treated at the Affiliated Hospital of Jiangnan University were reviewed retrospectively from January 2014 to 2020. CSP was classified into three types based on the location and shape of gestational tissue, blood flow features, and thickness of the myometrium at the incision site. The clinical characteristics, types, approaches of treatment, and clinical outcomes of CSP were analyzed.

**Results:** A total of 55 patients were included in this study, 29 (52.7%) of whom underwent transvaginal curettage after uterine artery embolization (UAE) and 22 (40%) of whom underwent transabdominal ultrasound-guided hysteroscopic curettage (USHC) in type I and II. Four patients (7.3%) classified as type III underwent laparoscopic cesarean scar resection (LCSR). Intraoperative blood loss, blood transfusion rate, and scar diverticulum were significantly higher in type II than in type I (*P* < 0.05). Even though USHC showed no differences in intraoperative blood loss, length of stay, and scar diverticulum compared with curettage after UAE (*P* > 0.05), superiority was found in surgical time and hospitalization cost (*P* < 0.05). Furthermore, the type of CSP (OR = 10.53, 95% CI: 1.69–65.57; *P* = 0.012) and diameter of the gestational sac (OR = 25.76, 95% CI: 2.67–248.20; *P* = 0.005) were found to be risk factors for intraoperative hemorrhage.

**Conclusions:** Transabdominal ultrasound-guided hysteroscopic curettage is an effective and relatively safe treatment option for patients with CSP. Type of CSP and diameter of the gestational sac were found to be associated with excessive intraoperative hemorrhage.

## Introduction

Cesarean scar pregnancy (CSP) is a rare form of ectopic pregnancy where the gestational sac, villi, and placenta are wholly or partially implanted in the myometrium of a previous scar (1). The incidence of CSP has been reported as approximately 1:2,000 of all pregnancies (2, 3). With the increasing number of cesarean sections (CS) being performed, regardless of the indication, the morbidity associated with CSP has shown a clear increasing global trend (4, 5).

Most patients with CSP show no specific symptoms and diagnosis depends on a recognition of the gestational sac at the uterine incision, as identified by ultrasound or magnetic resonance imaging (MRI). However, due to the relatively low sensitivity of imagological examinations, many CSP patients can be misdiagnosed. These misdiagnoses may lead to sharp curettage for a presumed failed pregnancy and result in major complications (6).

There is no worldwide consensus on the treatment of CSP (7). Treatment options include expectant management, medication, uterine artery embolization (UAE), curettage, surgery, and their combination (8), but the optimal management remains to be determined. Improper management may lead to severe complications, such as life-threatening hemorrhage, uterine rupture, hysterectomy, and even death (9, 10). To solve this problem, research is currently ongoing to find promising treatment methods for different types of CSP, that will reduce the incidence of severe bleeding, thus preserving the uterus and fertility.

Over the last decade, various studies have examined the risk factors associated with intraoperative bleeding in CSP. Research indicates that the diameter of the gestational sac and gestational age were considered to be likely risk factors for heavy bleeding during CSP treatment (11). Myometrial thickness, peritrophoblastic perfusion, and serum β-HCG have also been reported as risk factors of heavy bleeding (12, 13). These studies show that intraoperative bleeding was not related to age, parity, and gravidity; whereas conclusions regarding serum β-HCG and fetal heartbeat are inconsistent. There is significant heterogeneity in the treatments of CSP and the cutoff values for each parameter in such research. Furthermore, in a recent meta-analysis (14), multiple gravidities, large gestation sac, advanced gestational days, high serum β-HCG level, abundant blood supply to the pregnancy sac, and a thin myometrium were found to be risk factors for intraoperative bleeding in CSP. In consideration of the limitations of the model, their data was more conservative, there may be other risk factors that have not been explored. It is therefore still necessary to explore factors associated with intraoperative hemorrhage in CSP.

In this study, we presented the clinical characteristics of CSP, compared different treatments, and analyzed the risk factors for intraoperative hemorrhage with the aim of providing constructive information for dealing with this disease.

## Methods

### Patients

We studied CSP cases diagnosed and treated in the Affiliated Hospital of Jiangnan University from January 2014 to 2020, retrospectively. The age, body mass index (BMI), gravidity, parity, number of previous CS, gestational age, time of interval between last pregnancy and cesarean scar pregnancy, the diameter of the gestational sac, fetal heartbeat, and β-HCG were analyzed by reviewing the medical records shown in Table 1.

**Table 1:** Clinical characteristics of the 55 CSP patients involved in the current study.

**Characteristic**	**Value (*n* = 55)**
Age (years)	32.55 ± 5.39
BMI (kg/m^2^)	22.82 ± 2.86
Gravidity	3.51 ± 1.64
**Parity**	
1	45 (81.8%)
2	8 (14.5%)
3	2 (3.6%)
**Number of previous CS**	
1	49 (89.1%)
2	6 (10.9%)
**Presenting symptoms**	
Abdominal pain	11 (20.0%)
Vaginal bleeding	20 (36.4%)
None	31 (56.7%)
GESTATIONAL age (weeks)	7.98 ± 2.51
Interval between previous CS and present (years)	6.58 ± 5.12
Diameter of gestational sac (mm)	34.51 ± 17.31
**Fetal heartbeat**	
Yes	12 (21.8%)
No	43 (78.2%)
Blood β-hCG (mlU/mL)	24,891 (619.8, 118,823)
Surgical time (min)	59.45 ± 26.35
**Imaging diagnosis**	
Ultrasound	28 (50.9%)
Ultrasound and MRI	27 (49.1%)
**Type of CSP**	
I	23 (41.8%)
II	28 (50.9%)
III	4 (7.3%)

### Ethical Approval and Consent to Participate

All procedures performed in this study involving human participants were in accordance with the ethical standards of the institutional and/or national research committee and with the 1964 Helsinki declaration and its later amendments or comparable ethical standards. The study design was approved by the Ethics Council of the Affiliated Hospital of Jiangnan University. Written informed consent was obtained from individual or guardian participants and stored in the Medical Records Room of the Affiliated Hospital of Jiangnan University.

### Diagnosis

The diagnosis of CSP mainly depends on transvaginal sonography (TVS) with a positive pregnancy test. The diagnostic criteria were as follows: (1) an empty uterine cavity or cervical canal with a clearly visible endometrium; (2) a gestational sac or mixed-echo mass located in the anterior isthmus or in the cesarean scar defect; (3) diminished myometrium between the bladder wall and the sac or the mass, or a discontinuity in the anterior uterine muscular tissues (15); (4) doppler examination showing high-velocity, low-impedance peritrophoblastic flow surrounding the gestational sac; and (5) the gestational sac did not slide following the application of gentle pressure to the cervix (3). Some CSP patients screened using TVS also underwent MRI examination.

### CSP Types

CSP was reclassified into three types (shown in Table 2), according to the Family Planning Group, the Chinese Medical Society of Obstetrics and Gynecology Expert Consensus on Diagnosis and Treatment of Cesarean Section Scar Pregnancy (16), which has been previously described in detail (17). The classification method is based on the location and shape of gestational tissues, blood flow features, and, most importantly, the myometrial thickness at the incision region demarcated by 3 mm.

**Table 2:** CSP classification according to the Family Planning Group, the Chinese Medical Society of Obstetrics, and Gynecology Expert Consensus on Diagnosis and Treatment of Cesarean Section Scar Pregnancy.

**Type**	**Diagnostic standards**
I	(1) Gestational tissue was partially implanted in the uterine scar, partially or mostly located in the uterine cavity, and some even reached the bottom of the uterine cavity; (2) The gestational sac was obviously deformed, elongated, and the lower end was acute; (3) The myometrium between the gestational sac and the bladder was >3 mm; (4) CDFI: trophoblast blood flow signal (low resistance blood flow) was seen in the scar.
II	(1) Gestational tissue was partially implanted in the uterine scar, partially or mostly located in the uterine cavity, and some even reached the bottom of the uterine cavity; (2) The gestational sac was obviously deformed, elongated, and the lower end was acute; (3) The myometrium between the gestational sac and the bladder was ≤3 mm; (4) CDFI: trophoblast blood flow signal (low resistance blood flow) was seen in the scar.
III	(1) Gestational tissues were completely implanted in the muscular layer of the uterine scar and protruded outward to the bladder; (2) Emptiness of the uterine cavity and cervical canal; (3) The myometrium between the gestational sac and the bladder was significantly thinner or even missing, the thickness was ≤3 mm; (4) CDFI: trophoblast blood flow signal (low resistance blood flow) was seen in the scar.

### Surgical Approach

The surgical approach was determined based on a combination of the patient's wishes, indications and contraindications for surgery, and a clinician's recommendations. The surgical treatments included transvaginal curettage after UAE for 29 cases, transabdominal ultrasound-guided hysteroscopic curettage (USHC) for 22 cases, and laparoscopic cesarean scar resection (LCSR) for four cases.

#### Curettage After UAE

Curettage was performed 1–3 days after UAE by the interventional surgeon. The venous passage was established, and the other rescue equipment was prepared. Curettage was performed by a highly experienced gynecologist in the Affiliated Hospital of Jiangnan University. Tissue and intraoperative blood loss were recorded during surgery. If the villus was not intact, the tissues of pregnancy would need to be confirmed by pathologists.

#### USHC

Patients underwent hysteroscopic surgery with ultrasonographic guidance performed by experienced gynecologists and sonographers. Under epidural anesthesia and after routine disinfection, cervical dilatation was carefully and successively achieved using dilators. Subsequently, an operative hysteroscope was placed inside the uterus.

The ectopic gestational tissue was directly visible under hysteroscopy. The vessel bed at the implanted site was exposed and coagulated for hemostasis. Hysteroscopic surgery was performed under ultrasonographic monitoring, and larger tissues were removed with a pair of oval forceps. Then, the residual gestational tissue was excised to the superficial muscle using an electric loop. During the process of electrosurgical excision, blood flow was immediately stopped by electrocoagulation, and the excised tissues were sent for pathological examination. Finally, the uterine scar diverticulum was checked thoroughly to confirm that there was no obvious residual tissue or active bleeding. Dynamic ultrasound can be used to observe blood flow at any time to ensure the complete removal of all gestational tissue. On the other hand, a static ultrasound can be used to measure residual scar thickness to prevent uterine perforation.

#### LCSR

The process of LCSR is similar to general laparoscopy. First, the bladder, uterus, and reflexive peritoneum were revealed, and the bladder was pushed down to expose the isthmus region of the uterus. Then, moderate pituitrin diluted with saline was injected to determine the site of the CSP lesions, and the entire scar area, including the pregnancy tissue, was removed with an ultrasound knife. Next, the uterine defect was rinsed and trimmed and the wound was modified and sutured for two layers. Finally, a hysteroscopy was performed to ensure that the lesions were completely excised and that the incision was well-aligned.

### Assessment of Intraoperative Blood Loss

The intraoperative bleeding volume was measured by the weight change of gauzes and operator estimation. Excessive intraoperative hemorrhage was defined as bleeding ≥ 200 mL.

### Statistical Analysis

All of the data were analyzed using SPSS 23.0 (SPSS, Inc., USA). A two-tailed significance test was used for all comparisons, and statistical significance was set at *P* < 0.05. The measurement results were shown as mean ± standard deviation (mean ± SD). Categorical data were reported as *n* (%) and compared using the Pearson Chi-square test, continuity correction, or Fisher's exact test. Univariate and multivariate (if found to be statistically significant in univariate analysis) logistic regression models were established to estimate the association between candidate risk factors and excessive intra-operative hemorrhage, and the results are shown as Odds ratios (ORs) with their 95% confidence intervals (CIs).

## Results

This study retrospectively analyzed 55 CSP patients who had an average age of 32.55 ± 5.39 years, ranging from 22 to 44 years, and average previous parity of 3.51 ± 1.64, ranging from 1 to 6. All of the patients had a history of CS in their lower uterine segments, regardless of the indication. Among them, 49 patients underwent CS once and 6 underwent the procedure twice. The average time since last CS was 6.58 ± 5.12 years, and the mean gestational age was 7.98 ± 2.51 weeks. The diameter of the gestational sac ranged from 9 to 97 mm with an average of 34.51 ± 17.31 mm. Blood β-HCG levels ranged from 619.8 to 118,823 mIU/mL with a median of 24,891 mIU/mL. Twenty patients (36.4%) had vaginal bleeding, and 11 patients (20%) had abdominal pain, whereas 31 (56.7%) cases had no symptoms and were diagnosed by TVS. In total, 23 cases (41.8%) were diagnosed with type I, 28 (50.9%) cases were diagnosed with type II, and 4 (7.3%) were diagnosed with type III (shown in Table 1).

In the subgroup analysis, which compared two surgical approaches, USHC was as safe and effective as curettage after UAE but proved to be more economical. Among 29 patients treated with curettage after UAE, 15 (51.7%) patients were classified as type I, and 14 (48.3%) patients were classified as type II. In total, 22 cases underwent USHC, of which 8 (36.4%) cases were diagnosed as type I and 14 (63.6%) patients were diagnosed as type II. Differences in the CSP classification between the treatments were not significant (*P* = 0.275). With regard to the outcomes of the two approaches, there were no significant differences in intraoperative blood loss and blood transfusion (*P* > 0.05; see Table 3). For supplementary treatments, which were defined as therapy after the primary process, only eight cases (4.5%) in the USHC group needed methotrexate (MTX) chemotherapy by intramuscular injection, whereas five cases (17.2%) in curettage after UAE were injected with MTX, and one case (3.4%) underwent secondary curettage and balloon compression. No significant difference was shown in the rate of supplementary treatment between the two treatments in this study. Regarding short-term hospitalization, there was no significant difference in length of stay between the two methods (*P* = 0.735), but the USHC group had shorter surgical time and was less costly (*P* < 0.05). Moreover, in terms of long-term follow-up, there was no significant difference between the two methods in results of the uterine scar diverticulum (*P* > 0.05; see Table 3).

**Table 3:** Comparison between surgical approaches.

**Feature**	**Curettage after UAE (*n* = 29)**	**USHC (*n* = 22)**	***P***
**CSP type**			0.275^*^
I	15 (51.7%)	8 (36.4%)	
II	14 (48.3%)	14 (63.6%)	
**Intraoperative blood loss**			0.906^*^
<200 ml	22 (75.9%)	17 (77.3%)	
≥200 ml	7 (24.1%)	5 (22.7%)	
Blood transfusion	4 (13.8%)	1 (4.5%)	0.375^***^
Supplementary treatment	6 (20.7%)	1 (4.5%)	0.124^***^
Surgical time	62.93 ± 15.21	45.45 ± 19.27	0.001^**^
Length of stay	6.48 ± 3.02	6.23 ± 2.07	0.735^**^
Expenses	15,364.52 ± 2,799.40	12,571.77 ± 3,649.09	0.003^**^
Scar diverticulum	12 (41.4%)	12 (54.5%)	0.351^*^

* *Chi-square*,

** *Student's t-test*,

*** *Fisher's exact test*.

Because all of the four patients diagnosed with type III received LCSR only, their clinical characteristics and outcomes were listed in Table 4 and analyzed separately. All four patients had undergone only one CS and they were at least 9 weeks gestational age before they were first diagnosed. The diameter of gestational sacs ranged from 45 to 97 mm, and plasma β-hCG ranged from 19,152 to 46,059 mlU/mL. A fetal heartbeat was only detected in one patient. As a result, three patients had intraoperative bleeding of more than 1,000 ml and received a blood transfusion. All four cases were successfully excised without supplementary treatment. No incidence of hysterectomy was recorded.

**Table 4:** Clinical characteristics of patients with type III who underwent LCSR.

**Case No**.	**Age (years)**	**Gravidity and parity**	**Number of previous cesarean sections**	**Interval time (years)**	**Gestational age (week)**	**Diameter of gestational sac (mm)**	**Fetal heartbeat**	**Blood β-hCG (mlU/mL)**	**Surgical time (min)**	**Intraoperative blood loss (mL)**	**Blood transfusion (mL)**	**Length of stay (d)**	**Expenses (CNY)**
1	28	G2P1	1	3	9.0	45	Yes	27,471	175	2,000	1,400	10	26,579
2	29	G4P2	1	5	12.9	97	No	19,152	90	2,800	1,800	12	30,002
3	27	G6P1	1	5	9.0	45	No	46,059	130	1,000	800	7	23,286
4	27	G5P1	1	5	10.3	54	No	40,651	70	500	–	6	18,010

Finally, we analyzed risk factors for intraoperative hemorrhage for CSP. Table 5 lists the results of univariate analysis and multivariable analysis of risk factors for intraoperative hemorrhage in CSP patients. In single-variable statistical analyses, the type of CSP and diameter of the gestational sac revealed statistically significant differences between high and low blood loss groups (*P* < 0.05); similar results appeared on multivariable logistic regression analysis (*P* < 0.05).

**Table 5:** Univariate and multivariable analysis of risk factors associated with intraoperative hemorrhage in CSP patients.

**Variable**	**Univariate**		**Multivariate**	
	**OR (95% CI)**	***P***	**OR (95% CI)**	***P***
Age ≥ 33 years	0.55 (0.15,2.05)	0.371^*^	–	–
**Parity**				
1	0.35 (0.04, 3.15)	0.593^***^	–	–
2	–	0.271^***^	–	–
3	0.29 (0.02, 5.01)	0.419^**^	–	–
Number of previous CS	–	0.315^**^	–	–
Gestational age > 7 weeks	1.47 (0.47, 5.45)	0.560^*^	–	–
Interval between previous CS and present ≥ 7 years	0.53 (0.53, 2.29)	0.612^***^	–	–
Fetal heart beat	1.29 (0.28,5.91)	1.000^***^	–	–
Surgery approach	0.92 (0.25,3.43)	0.906^*^	–	–
Type of CSP	5.83 (1.13,30.18)	0.024^*^	10.53 (1.69, 65.57)	0.012
β-HCG ≥ 20,000 mlU/mL	2.32 (0.54, 9.90)	0.415^***^	–	–
Diameter of gestational sac ≥ 35mm	15.81 (1.85,134.97)	0.002^*^	25.76 (2.67, 248.20)	0.005

* *Chi-square*,

** *Fisher's exact test*,

*** *Continuity correction*.

## Discussion

Since first reported in 1978 (18), CSP has been regarded as one of the long-term risks of cesarean delivery. The number of CSPs reported has increased with the increasing number of CS being performed globally and the wide use of TVS for screening (19). Patients primarily complain of vaginal bleeding and sometimes of abdominal pain, but the symptoms are nonspecific. Therefore, early diagnosis of CSP is difficult. The disease has often been misdiagnosed as threatened abortion, incomplete abortion, cervical pregnancy, malignant trophoblastic tumor, and so on. Such misdiagnoses may lead to uncontrollable vaginal bleeding caused by inappropriate induction of abortion or curettage (20).

CSP can be classified as either an endogenic type or an exogenic type. With the former, a pregnancy may be left to continue providing the patients are counseled and willing to undertake the potential risk of massive hemorrhage (21, 22). However, this classification is not conducive to the clinical situation because it lacks quantitative indicators that can be used to guide clinical treatment. The Family Planning Group, Chinese Medical Society of Obstetrics and Gynecology proposed to divide CSP into three types on the basis of location and shape of the gestational tissue, myometrial thickness, and blood flow in the incision region (16); this new classification was used in this study. Type I and II patients were treated with curettage after UAE or USHC, whereas type III patients received LCSR. Only seven cases needed supplementary treatment, and no patients underwent a hysterectomy, indicating that the classification was more precise and provided superior treatment guidance.

There is currently no consensus on the optimal management of CSP worldwide. The treatment options are predominantly expectant management, medical, or surgical. Expectant management may be acceptable in CSP patients with no fetal cardiac activity, but there is a very high risk for placenta accreta syndrome. In CSP patients with fetal cardiac activity, even though ~3 quarters achieve live births, a significant number of them face uterine rupture, severe bleeding, and subsequent hysterectomy (6, 23). The medical management of CSP mainly involves MTX and this shows better outcomes than expectant treatment. Local MTX injection guided by TAS (transabdominal sonography) or TVS is recommended as the first-line treatment for CSP patients with HCG levels <100,000 IU/L because of its relatively high success rate (73.9% for single and 88.5% for multiple injections) and low complication rate (about 9.6%) (24, 25). Surgery may also be considered a good choice in the treatment of CSP. A national cohort study in the UK that was performed recently shows surgery to be associated with a high success rate (96%), low complication rate (36%), and short post-treatment follow up (19).

Our study explored the appropriate surgical approach for patients with CSP. Three methods, including curettage after UAE, USHC, LCSR, were used in the CSP patients. For type III patients, whose myometrial thickness at the incision site was thin, general uterine curettage may increase the risk of residual and uterine perforation. LCSR is suitable for patients where lesions grow toward the outside of the uterus because removal of the lesion and uterine repair can be performed simultaneously (20). Thus, all type III cases underwent LCSR in this study and achieved successful outcomes. The result here was in accordance with the past studies (8, 26), which found that LCSR were associated with a high success rate (95.5–97.1%) and no major complications for all types of CSP. However, it may be over treatment if all the patients (including the type I and II patients) choose LCSR firstly. So, we propose that type III may be a good indication and deserve further research.

For type I and II patients, there was no significant difference in the constituent ratio of the two methods: UAE and USHC. UAE is a useful technique for hemostasis and preservation of the uterus (27); it blocks the blood supply of the uterine artery and branches, effectively reducing blood loss and blood flow intra- and post-surgery (28). It is reported that the success rate of dilatation and curettage combined with UAE achieved 94.5–100% in CSP. Despite its extensive use in CSP, UAE is not favorably recommended as the first-line strategy for women who are planning on future pregnancies. Previous studies have shown an adverse effect on endometrial and ovarian function of UAE (29, 30). Hysteroscopic removal of CSP has been reported to be safe and effective as an alternative minimally invasive surgery (31). The advantage of hysteroscopic removal is that resection can be carried out under direct vision. With ultrasound surveillance, the uteroscope entered the uterus via the vagina, and the tissues could be visually identified. This can avoid lesions or extensive damage to the endometrium caused by curettage (32), to maximize the preservation of fertility. Our data showed that the USHC group had a shorter surgical time and a lower hospital cost than the curettage for the UAE group, but it was not inferior in reducing intraoperative blood loss, supplementary treatment rate, length of stay, or scar diverticulum rate. Moreover, only one case needed supplementary treatment after USHC. These results demonstrate the impressive security, effectiveness, and economy of USHC. Thus, USHC is an optional approach in the treatment of type I and II CSP. It should be noted that surgical skill also played an important role; the choice of surgery must be individualized according to the surgeon's experience and the patient's choice.

It is very interesting that the USHC group had lower hospital costs, as there was no difference in hospitalization time. The cost advantage may have come from avoiding the use of expensive UAE supplies, as well as the reduced surgical time and hence the cost of anesthesia and monitoring.

We further analyzed the potential risk factors for excessive intraoperative hemorrhage, including age, parity, β-HCG, number of previous CS, gestational age, interval between previous CS and present, fetal heartbeat, diameter of gestational sac, surgical approach, and the type of CSP. We found that severe intraoperative blood loss was correlated with the diameter of the gestational sac and the type of CSP. The above indexes can help filter those patients with a high risk of intraoperative hemorrhage and select treatment options for them. However, the present study had shortcomings, such as the small sample size and retrospective analysis. Thus, the conclusions of this study need to be further investigated with an increased number of cases and the use of randomized clinical trials.

In conclusion, transabdominal ultrasound-guided hysteroscopic curettage is an effective and relatively safe treatment option for patients with CSP. Treatment should be personalized according to the actual situation. Type of CSP and diameter of gestational sac were found to be associated with excessive intraoperative hemorrhage.

## Data Availability Statement

The data used and analyzed during the current study are available from the corresponding author on reasonable request.

## Author Contributions

YL, CX, CD, and JY designed the study, collected and analyzed the data, wrote the manuscript, and were involved in revising the manuscript. All authors have approved the final version.

## Conflict of Interest

The authors declare that the research was conducted in the absence of any commercial or financial relationships that could be construed as a potential conflict of interest.
